# Supplementary material for: Determinants of Allergic Sensitization, Asthma and Lung Function: Results from a Cross-Sectional Study in Italian Schoolchildren
Source: Int J Environ Res Public Health. 2020 Jul 14;17(14):5087. doi: 10.3390/ijerph17145087 (PMC7400051; doi:10.3390/ijerph17145087)
Supplement: Supplementary file 1 [file ijerph-17-05087-s001.pdf]

Supplementary material: Tables

Table S1 – Multiple linear regression model for $FEF_{25-75\%}$ (z-score), separately for female and male children. Significant *p* values are in bold.

A - Females

Independent variables	B	95% confidence interval		p value
		Lower	Upper	
Education level (Ref.: higher)	-0.077	-0.224	0.070	0.307
Exposed to maternal smoke during pregnancy (Ref.: not exposed)	-0.112	-0.340	0.116	0.338
Early exposure to environmental tobacco smoke (Ref.: not exposed)	-0.022	-0.201	0.157	0.811
Acute respiratory diseases in the first two years (Ref.: not reported)	-0.250	-0.413	-0.087	**0.003**
Nocturnal cough (Ref.: not reported)	0.033	-0.152	0.218	0.727
Current asthma (Ref.: not reported)	-0.430	-0.847	-0.014	**0.043**
Exercise wheezing (Ref.: not reported)	-0.081	-0.449	0.288	0.669
Rhinoconjunctivitis (Ref.: not reported)	-0.068	-0.305	0.170	0.577
Current exposure to environmental tobacco smoke (Ref.: not reported)	0.027	-0.131	0.185	0.740
Residence in proximity to roads with intense vehicular traffic (Ref.: not reported)	0.145	-0.030	0.320	0.105
Mould/dampness at home (Ref.: not reported)	0.132	-0.103	0.367	0.270
Pet ownership (Ref.: not reported)	0.080	-0.084	0.244	0.339
Allergic sensitization (Ref.: negative)	0.038	-0.114	0.190	0.627
Weight (kg)	0.006	0.000	0.012	**0.036**

B - Males

Independent variables	B	95% confidence interval		p value
		Lower	Upper	
Education level (Ref.: higher)	0.064	-0.074	0.203	0.365
Exposed to maternal smoke during pregnancy (Ref.: not exposed)	-0.428	-0.650	-0.205	**<0.0001**
Early exposure to environmental tobacco smoke (Ref.: not exposed)	0.224	0.055	0.393	**0.010**
Acute respiratory diseases in the first two years (Ref.: not reported)	-0.297	-0.443	-0.151	**<0.0001**
Nocturnal cough (Ref.: not reported)	-0.007	-0.174	0.160	0.931
Current asthma (Ref.: not reported)	-0.445	-0.884	-0.006	**0.047**
Exercise wheezing (Ref.: not reported)	-0.046	-0.417	0.326	0.809
Rhinoconjunctivitis (Ref.: not reported)	-0.162	-0.387	0.062	0.157
Current exposure to environmental tobacco smoke (Ref.: not reported)	0.089	-0.060	0.237	0.242
Residence in proximity to roads with intense vehicular traffic (Ref.: not reported)	0.036	-0.123	0.194	0.661
Mould/dampness at home (Ref.: not reported)	-0.065	-0.285	0.154	0.559
Pet ownership (Ref.: not reported)	-0.019	-0.191	0.153	0.828
Allergic sensitization (Ref.: negative)	0.131	-0.008	0.269	0.064
Weight (kg)	0.002	-0.003	0.007	0.507

Table S2 – Multiple linear regression model for $FEF_{25-75\%}$ (z-score) among 1,102 "healthy" subjects only*. Significant *p* values are in bold.

Independent variables	B	95% confidence interval		p value
		Lower	Upper	
Sex (Ref.: female)	0.085	-0.033	0.203	0.156
Education level (Ref.: higher)	0.050	-0.071	0.171	0.420
Maternal smoke during pregnancy (Ref.: not exposed)	-0.278	-0.481	-0.076	**0.007**
Early exposure to environmental tobacco smoke (Ref.: not exposed)	0.074	-0.076	0.224	0.332
Acute respiratory diseases in the first two years (Ref.: not reported)	-0.263	-0.402	-0.123	**<0.0001**
Current exposure to environmental tobacco smoke (Ref.: not reported)	0.014	-0.116	0.143	0.834
Residence in proximity to roads with intense vehicular traffic (Ref.: not reported)	0.002	-0.141	0.145	0.977
Mould/dampness at home (Ref.: not reported)	0.052	-0.144	0.247	0.603
Pet ownership (Ref.: not reported)	-0.049	-0.190	0.092	0.498
Allergic sensitization (Ref.: negative)	0.152	0.028	0.275	**0.016**
Weight (kg)	0.004	0.000	0.009	0.060

*Healthy subjects were children without doctor diagnosed asthma and not reporting wheeze, nocturnal cough, exercise wheeze or rhinoconjunctivitis in the last 12 months.

Table S3 – Multiple linear regression model for $FEF_{25-75\%}$/FVC ratio. Significant *p* values are in bold.

Independent variables	B	95% confidence interval		p value
		Lower	Upper	
Education level (Ref.: higher)	-0.021	-0.047	0.004	0.101
Early exposure to environmental tobacco smoke (Ref.: not exposed)	0.026	-0.005	0.057	0.100
Acute respiratory diseases in the first two years (Ref.: not reported)	-0.055	-0.083	-0.027	**<0.0001**
Nocturnal cough (Ref.: not reported)	0.011	-0.020	0.043	0.482
Current asthma (Ref.: not reported)	-0.085	-0.161	-0.008	**0.030**
Exercise wheezing (Ref.: not reported)	-0.043	-0.110	0.023	0.201
Rhinoconjunctivitis (Ref.: not reported)	-0.043	-0.084	-0.002	**0.041**
Current exposure to environmental tobacco smoke (Ref.: not reported)	0.007	-0.021	0.034	0.627
Residence in proximity to roads with intense vehicular traffic (Ref.: not reported)	0.030	0.000	0.060	0.047
Mould/dampness at home (Ref.: not reported)	0.004	-0.037	0.044	0.860
Pet ownership (Ref.: not reported)	0.002	-0.028	0.032	0.895
Allergic sensitization (Ref.: negative)	0.022	-0.004	0.048	0.097
BMI (kg/m^2)	-0.011	-0.014	-0.008	**<0.0001**
Exposed to maternal smoke during pregnancy * male sex †	-0.189	-0.247	-0.131	**<0.0001**
Not exposed to maternal smoke during pregnancy * male sex †	-0.106	-0.134	-0.079	**<0.0001**
Exposed to maternal smoke during pregnancy * female sex†	-0.016	-0.072	0.041	0.587

†Ref.: not exposed to maternal smoke during pregnancy * female sex
